# Silk Fibroin/Spidroin Electrospun Scaffolds for Full-Thickness Skin Wound Healing in Rats

**DOI:** 10.3390/pharmaceutics13101704

**Published:** 2021-10-15

**Authors:** Liubov Safonova, Maria Bobrova, Anton Efimov, Lyubov Davydova, Timur Tenchurin, Vladimir Bogush, Olga Agapova, Igor Agapov

**Affiliations:** 1 Academician V.I. Shumakov National Medical Research Center of Transplantology and Artificial Organs, Ministry of Health of the Russian Federation, Schukinskaya ul. 1, 123182 Moscow, Russia; saf.lyubov.msu@gmail.com (L.S.); mariabobrova.msu@gmail.com (M.B.); antefimov@gmail.com (A.E.); olya.agape@gmail.com (O.A.); 2 National Research Center “Kurchatov Institute”, pl. Academician Kurchatov 1, 123182 Moscow, Russia; davidovlu@gmail.com (L.D.); tenchurin.timur@mail.ru (T.T.); vlbogush@mail.ru (V.B.)

**Keywords:** silk fibroin, recombinant spidroin, electrospinning, scanning probe nanotomography, full-thickness skin wound

## Abstract

The main goal of our research was to fabricate electrospun scaffolds from three different silk proteins—silk fibroin from *Bombyx mori* silkworm cocoons and two recombinant spidroins, rS2/12 and rS2/12-RGDS—and to perform a comparative analysis of the structure, biological properties, and regenerative potential of the scaffolds in a full-thickness rat skin wound model. The surface and internal structures were investigated using scanning electron microscopy and scanning probe nanotomography. The structures of the scaffolds were similar. The average fiber diameter of the scaffolds was 315 ± 26 nm, the volume porosity was 94.5 ± 1.4%, the surface-to-volume ratio of the scaffolds was 25.4 ± 4.2 μm^−1^ and the fiber surface roughness was 3.8 ± 0.6 nm. The scaffolds were characterized by a non-cytotoxicity effect and a high level of cytocompatibility with cells. The scaffolds also had high regenerative potential—the healing of the skin wound was accelerated by 19 days compared with the control. A histological analysis did not reveal any fragments of the experimental constructions or areas of inflammation. Thus, novel data on the structure and biological properties of the silk fibroin/spidroin electrospun scaffolds were obtained.

## 1. Introduction

One of the main objectives in tissue engineering is the fabrication of cytocompatible constructions and the selection of materials that can perform cell interactions to ensure the physiological activity of the construction. There is a spectrum of requirements for these materials, such as non-toxicity, a defined biodegradation rate, low immunogenicity, etc. The structure of the construction should imitate the native extracellular matrix structure as closely as possible and perform its functions to recreate the native conditions for cells.

This research investigated constructions that were fabricated from three different silk proteins: silk fibroin from cocoons of the *Bombyx mori* silkworm, and two recombinant spidroins rS2/12 and rS2/12-RGDS.

Silk fibroin is characterized by a unique combination of physico-chemical and biological properties, and can be used in different fields of tissue engineering, both in a solo state and in composites. The main advantage of silk when compared with other cytocompatible materials is its mechanical properties [1], which ensure the fibroin application as a frame-reinforcing component in various constructions [2,3] and as a composite additive to polymers with insufficient mechanical strength [4,5,6].

The primary structure of fibroin has the main repeating motif GAGAGS, which accounts for 95% of amino acid residues. The remaining part has an amorphous structure and consists mainly of hydrophilic amino acid residues. The secondary structure presents antiparallel β-layers connected by hydrogen bonds. The amorphous regions form α-helices, the proportion of which increases with protein hydration. The tertiary structure includes a heavy chain with a molecular weight of 390 kDa and a light chain of 26 kDa in a 1:1 ratio, connected by disulfide bonds, as well as a P25 glycoprotein of 30 kDa, which assemble in a 6:6:1 ratio correspondingly to form a complex [7].

Spidroins are proteins synthesized by spider glands. The formation and structuring processes of the main silk proteins of the orb-weaver spiders *Nephila clavipes* and *Araneus diatematus* have been studied extensively. The structural thread consists of two proteins, spidroin 1 and spidroin 2, which form a complex [8]. Both of these proteins are characterized by the presence of a huge number of repetitive sequences in the central part (the so-called primary repeats of 25–40 amino acid residues in size) and unique sequences of 100–300 amino acid residues at the N- and C-domains. All repeats contain poly-Ala blocks in 4–8 amino acid residues, which alternate with Gly replete regions with the GGX motif for spidroin 1 and the GPGXX motif for spidroin 2. Such an alteration of the hydrophobic and hydrophilic regions of molecules ensures amphiphile properties for interaction with tissues. The presence of up to 15% of proline residues in the amino acid sequence of spidroin 2, which are absent in spidroin 1 [9], has a significant effect on the further formation of higher-level structures and determines the various properties of these proteins.

Similar to silk fibroin, spidroins are characterized by the ability to phase transition during dehydration. This property makes it possible to ensure the structural stability of the protein in constructions that are based on them.

The attention of researchers on spider proteins is primarily due to the unique mechanical characteristics of these proteins. The tensile strength of spidroins is comparable to the tensile strength of Kevlar, while the elasticity of spidroin is about seven times higher, leading to impressive energy of break values [10,11]. Such mechanical characteristics make it possible to consider spidroins as promising materials for use in technology. However, the use of spidroins is still limited by the difficulties associated with obtaining these proteins on an industrial scale.

The development of genetic engineering has made it possible to create recombinant analogues of spidroins that not only have unique mechanical characteristics, but are also cytocompatible, making it possible to consider these spidroins as promising material for the development of tissue-engineered constructions [12,13]. Genetic engineering also makes it possible to create modified forms of recombinant spidroin analogues with a set of properties that correspond to a required task.

On the basis of recombinant spidroins, various materials have been created, such as hydrogels and microgels, porous scaffolds, tubes, scaffolds, films, etc. [14,15,16,17,18,19,20].

The electrospinning method is one of the most promising methods for fabricating scaffolds with a defined structure. Electrospun scaffolds have a multilayer fibrous structure with a high porosity and a high surface area-to-volume ratio (SA:V). Many different types of constructions based on silk proteins have been fabricated using the electrospinning method [17,21].

Using a mixture of fibroin and recombinant spidroins makes it possible to create cytocompatible constructions that combine mechanical properties and high cytocompatibility with modification potential. These properties allow the requirements of tissue engineering to be satisfied. Furthermore, fibroin and spidroins are characterized by high strength and an elasticity modulus, which are necessary to accelerate regenerative potential and to reduce surgical trauma.

Thus, in the course of this study, a comparative analysis of the structure, biological properties and regenerative potential of silk fibroin/spidroin electrospun scaffolds was performed and novel data on its structure and biological properties was obtained.

## 2. Materials and Methods

### 2.1. Preparation of Silk Proteins

Silk fibroin was obtained from *B. mori* silkworm cocoons [22], which were provided by the head of the State Scientific Institution of the Republican Scientific Research Station of Sericulture of the Russian Academy of Agricultural Sciences (Zheleznovodsk, Stavropol Krai, Russia) Bogoslovsky V.V. To purify the cocoons from sericin, 1 g of silk was boiled in a water bath for 40 min in 500 mL of 2.52 M sodium bicarbonate aqueous solution and washed with 3.6 L of distilled water. Next, the silk was boiled in 500 mL of distilled water in a water bath for 30 min and washed with 3.6 L of distilled water. The last procedure was repeated three times. The purified silk fibroin was dried at room temperature.

Recombinant spidroins rS2/12 and rS2/12-RGDS were isolated from the water-insoluble fraction of yeast cell producers and purified by ion-exchange chromatography in an FPLC (Fast protein liquid chromatography) system up to 95% purity and lyophilized as described earlier [18].

### 2.2. Fabrication of Silk Fibroin-Based Microfibrous Scaffolds

An aqueous solution of silk fibroin was dried in a Petri dish at room temperature. The dried silk fibroin was dissolved in hexafluoro-2-propanol (HFIP) at a rate of 50 mg/mL.

Recombinant spidroins rS2/12 and rS2/12-RGDS were dissolved in HFIP at a rate of 50 mg/mL.

The solutions were centrifuged for 10 min at 12,100× *g* and then mixed in a volume ratio of 7:3, respectively, to a total protein concentration of 50 mg/mL.

Microfibrous scaffolds were fabricated using the electrospinning method. The solutions were deposited to the fixed collector surface (steel plate) under an electric field with a voltage of 6.8–7 kV through a 23 G needle. The solution feed rate was 0.1 mL/h, and the needle–collector distance was 7 cm. The scaffolds were dried at room temperature for 2 days, and were then separated. To create scaffolds for cell adhesion and proliferation research, the solutions were deposited with similar parameters on cover glasses that were attached to the collector.

### 2.3. Surface Structure Analysis by Scanning Electron Microscopy (SEM)

The samples of the scaffolds were fixed with a 2.5% phosphate-buffered glutaraldehyde solution (pH 7.4) for 2 h at 4 °C in the dark, and were then washed with a phosphate-buffered saline. The samples were dehydrated by transfer through ethanol solutions with increasing concentrations of 10, 20, 50, 70, and 96% for 1 h in each concentration, and incubated in acetone for 30 min.

The samples were then exposed to critical point drying (31 °C, 72.8 kg/cm^2^) by the Quorum K850 Critical Point Dryer (Quorum Technologies, Lewes, UK). The dried samples were coated with a gold layer with a thickness of 10 nm in an argon atmosphere at 20 mA of ion current and 1 mbar of pressure by the Q150R ES rotary-pumped coating system (Quorum Technologies, Lewes, UK). The samples were analyzed using the Tescan Vega3 SBU scanning electron microscope (Tescan, Brno, Czech Republic) with an operating voltage of 15 kV. Imaging was performed by VegaTC software (Tescan, Brno, Czech Republic).

### 2.4. Analysis of the Construction Structure with Scanning Probe Nanotomography (SPNT)

The samples were fixed with a 2.5% phosphate-buffered glutaraldehyde solution (pH 7.4) for 2 h at 4 °C in the dark and washed with phosphate-buffered saline. The samples were dehydrated by transfer through ethanol solutions with increasing concentrations of 10, 20, 50, 70, and 96% for 1 h in each concentration. The samples were incubated in propylene oxide for 10 min twice and transferred into a mixture of epoxy medium and propylene oxide in a ratio of 1:1, then in a mixture of epoxy medium and propylene oxide in a ratio of 2:1. After that, the samples were incubated in an epoxy medium for 30 min at room temperature. Next, the samples were embedded into the epoxy medium and incubated at 45 °C for 24 h, and then for another 48 h at 60 °C.

The study was performed using the combined Ntegra Tomo system (NT-MDT Co., Zelenograd, Russia), which comprises a scanning probe microscope integrated with the ultramicrotome Leica EM UC6NT (Leica Microsystems GmbH, Vienna, Austria). Serial sections of the sample with a thickness of 150 nm were performed with the Diatome UltraAFM 45 diamond knife (Diatome, Nidau, Switzerland) on the ultramicrotome, with further measurements of the surface topography of each section taken with an atomic force microscope [23]. The measurements were performed in semi-contact mode with a scanning frequency of 1 Hz. Silicon cantilevers NSG10 with a resonant frequency of 240 kHz (NT-MDT, Zelenograd, Russia) were used for the measurements, with a tip curvature radius of no more than 10 nm.

Images were obtained, processed, and surface profiles constructed by Nova 1.0.26.1433 software (NT-MDT, Zelenograd, Russia). To assemble three-dimensional reconstructions, images were aligned at the scanning plane. The three-dimensional structures were analyzed using ImagePro AMS 6.0 software (MediaCybernetics Inc., Rockville, MD, USA), which includes the option of three-dimensional reconstruction.

### 2.5. Fourier-Transform Infrared Spectroscopy (FTIR) of Scaffolds

Spectra were recorded on a Nicolet iS5 (Thermo Scientific, Waltham, MA, USA) instrument using an iD5 ATR device (diamond, 4 cm^−1^ resolution, 32 scans). A deconvolutional analysis of the amide I band was carried out using Origin 2016 software. To reveal the features of the secondary structure of the protein, the Savitsky–Golay method smoothing procedure was used with polynomials of the second degree over 9 points. The detection of analytical signals in the form of peaks was carried out on the basis of an analysis of the characteristic points of the second derivative. The half-width of the peaks was recorded in the range from 10 to 30 cm^−1^ [24].

### 2.6. Cytotoxicity, Cell Adhesion, and Proliferation Tests

An analysis of cytotoxicity was performed using the MTT test [25]. The 3T3 mice fibroblasts were cultured in a 96-well plate in DMEM with a low concentration of glucose containing 10% fetal bovine serum, 0.324 mg/mL glutamine and 10 mg/mL gentamicin at 37 °C, 5% CO_2_ for 3 days. Next, the culture medium was changed and the scaffold samples were put into wells. The plates were incubated at 37 °C, 5% CO_2_. The MTT test was performed on the 3rd, 5th, and 7th days of the incubation. Per well, 60 μL of 5 mg/mL MTT solution was added. The plates were incubated at 37 °C, 5% CO_2_ for 4 h. The samples were removed and the plates were centrifuged for 5 min at 885× *g*. The formazan precipitate in each well was dissolved in 300 μL of dimethyl sulfoxide per well, and the optical density was measured at 540 nm.

To investigate cell adhesion and proliferative activity, a Hep-G2 cell line was used. The cells were cultured in DMEM with a high concentration of glucose containing 10% fetal bovine serum, 0.324 mg/mL glutamine and 10 mg/mL gentamicin at 37 °C, 5% CO_2_. The scaffolds on the cover glasses were positioned in Petri dishes with a diameter of 3.5 cm, sterilized with 70% ethanol for 30 min, and then irradiated with ultraviolet light for 30 min. Next, the scaffolds were washed three times with sterile phosphate-buffered saline and incubated in a culture medium for 30 min. The cell suspension in 4 mL of culture medium was transferred to Petri dishes at a rate of 20,000 cells per Petri dish and incubated at 37 °C, 5% CO_2_. Cell adhesion and proliferative activity were evaluated with the Carl Zeiss Axio Vert.A1 microscope (Zeiss, Jena, Germany). The samples were washed twice with phosphate-buffered saline and stained with DAPI fluorescent dye. An amount of 3 μg/mL of aqueous DAPI solution was added at a rate of 2 mL per Petri dish and incubated at 37 °C, 5% CO_2_ for 5 min. Subsequently, the samples were washed twice with phosphate-buffered saline. Cell images were obtained and processed with ZEN 2.3 (blue edition) software (Zeiss, Jena, Germany).

### 2.7. Full-Thickness Skin Wound Healing of Wistar Rats

Male Wistar rats weighing 200–300 g were used for the experiment. The rats were isolated from each other in single cages with free access to water and food. All animal experiments were performed in accordance with European Convention for the Protection of Vertebrate Animals Used for Experimental and other Scientific Purposes (ETS) and Directive 2010/63/EU, and approved by the Local Ethical Committee of the V.I. Shumakov Federal Research Center of Transplantology and Artificial Organs.

All operations on the animals were performed under inhalation ether anesthesia, which was provided with a desiccator at a rate of 50 mg/kg body weight. The animals were in spontaneous respiration with a frequency of 75 ± 10 respiratory cycles per minute, corresponding to the surgical stage of anesthesia.

The animals were divided into three groups (Table 1). In group 1 (control group), the same surgical procedures were performed on all the animals, and the wounds were covered with sterile gauze dressings.

The modeling of a full-thickness skin wound was performed as follows [26]. Hairs from the back of a rat were removed, and the skin was treated with a 0.05% chlorhexidine solution. Next, a wound with a diameter of 15 ± 1 mm was made and treated with a 0.05% chlorhexidine solution. The depth of the damage corresponded to the thickness of the rat skin, including epidermis, dermis and hypodermis.

Scaffolds were sterilized for 1 h with an ultraviolet light and placed on the wound area in a dry form. Next, the dressing wound was treated with a 0.05% chlorhexidine solution and coated with sterile gauze dressing, which was removed on the third day of the experiment.

The diameter of the wound (d) was measured and the wound healing area (A) was calculated on 0th, 3rd, 7th, 14th, 21st, 23rd, 28th, and 40th days of the experiment according to the formula:

(1) $$A=\frac{d_{(0)\;}{-\;d}_{\left(0,3,7,14,21,23,28,40\right)}}{d_{\left(0\right)}}\;\times \;100\%$$

A—wound healing area,

$d_{\left(0\right)}$—initial wound diameter (day 0),

$d_{\left(0,3,7,14,21,23,28,40\right)}$—wound diameter on the control day of the experiment.

Subsequently, the curves of the dynamics of the wound closure process were plotted.

### 2.8. Histological Evaluation

The samples of rat skin that were 20 ± 3 mm in size were fixed by a mixture of formalin, ethanol and acetic acid in a 4:1:0.3 volume ratio and were embedded into paraffin. The paraffin blocks were sliced; sections with a thickness of 10 μm were obtained using a Microm HM 325 rotary microtome (Thermo Scientific, Waltham, MA, USA), stained with hematoxylin-eosin, embedded in Canada balsam and analyzed using a Carl Zeiss Axio Vert.A1 microscope (Zeiss, Jena, Germany). Images were obtained with a Axiocam 305 color digital camera (Zeiss, Jena, Germany) and processed using ZEN 2.3 (blue edition) software (Zeiss, Jena, Germany).

### 2.9. Statistical Processing of Results

Data were processed using analysis of variance (ANOVA). The statistical significance of the results was evaluated by the Mann–Whitney U test. The level of statistical significance α was equal to 0.05.

## 3. Results and Discussion

**Figure 1:**
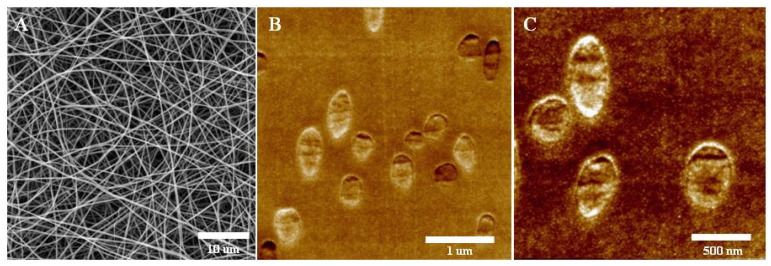
Structure of MS-RGD scaffolds: (**A**)—SEM image, (**B**,**C**)—SPM image. Images of MS scaffolds are not presented due to the absence of significant structural differences.

In the course of the study, two groups of scaffolds based on a mixture of fibroin and two variants of recombinant spidroin were fabricated using an electrospinning process (Figure 1A). Spidroin rS2/12 was included in the scaffolds of the first group, and spidroin rS2/12, containing at the C-terminus the amino acid sequence RGDS attached via a linker of 10 amino acid residues (GGSGG SGGSGG), was included in the scaffolds of the second group [12,13]. The RGDS amino acid sequence was introduced into the molecule in order to improve the adhesive properties of the constructions that were based on it, and to increase their cytocompatibility. The amphiphilic properties of these proteins contributed to the same goals, as did their positive charge over the entire range of physiological pH values (pI 10.13 for both types of spidroins). The inclusion of spidroins in the composition of scaffolds makes it possible to increase their cytocompatibility without reducing their mechanical characteristics (in particular, mechanical strength and elasticity), which is an advantage for scaffold implantation as it facilitates surgical procedures.

To fabricate the scaffolds, the electrospinning method was used as it allows for obtaining constructions with a high surface area-to-volume ratio, as well as those with a high porosity index. These structure properties ensure its closeness to the native extracellular matrix structure, providing its high cytocompatibility.

The structure of the fabricated scaffolds was analyzed using two methods. The SEM method (Figure 1A) made it possible to confirm the porous fibrous structure of the scaffolds, as well as to estimate the average thickness of the fibers in their composition, which was 328 ± 57 nm. The fibers in the scaffolds did not have a specific orientation and were stacked in several layers. To analyze the structure of the scaffolds with a high resolution and to estimate the quantitative parameters of the structure, the SPNT method was used (Figure 1B,C). Scanning probe microscopy (SPM) images were obtained to reflect the distribution of fibers in the volume of the scaffold (Figure 1B), and SPM images of the structure of the individual fibers were also obtained (Figure 1C). Here, we performed SPM measurements of the epoxy embedded scaffold blockfaces after section by the ultramicrotome with use of Scanning Probe Nanotomography (SPNT) technique and analyzed distribution of the cross-sections of the electrospun fibers. The images were used to calculate the crucial quantitative parameters of the structure of the fabricated scaffolds. The average fiber diameter in the scaffolds was 315 ± 26 nm, the volume porosity of the scaffolds was 94.5 ± 1.4%, the ratio of the fiber surface to the scaffold volume was 25.4 ± 4.2 μm^−1^ and the fiber surface roughness was 3.8 ± 0.6 nm. The calculated parameters were comparable for both groups of scaffolds.

Within the framework of this study, the quantitative parameters of the structure of the fabricated MS and MS-RGD scaffolds were for the first time estimated using the unique technology of SPNT. This technology allows the high-resolution visualization of the structure of sample surface after ultrathin sections and the calculation of their three-dimensional structure parameters. This method makes it possible to precisely determine the value of the scaffold fiber thickness and to confirm the uneven distribution of the fibers in the scaffold volume, as well as to calculate the crucial parameters of the structure [27,28,29]. Scaffolds have a high porosity index (over 90%) and a high surface area-to-volume ratio, which ensures the efficient migration of cells in the volume of scaffolds and provides a microenvironment for their adhesion. Fibers in scaffolds have a nanorelief on the surface, which also affects cell adhesion.

**Figure 2:**
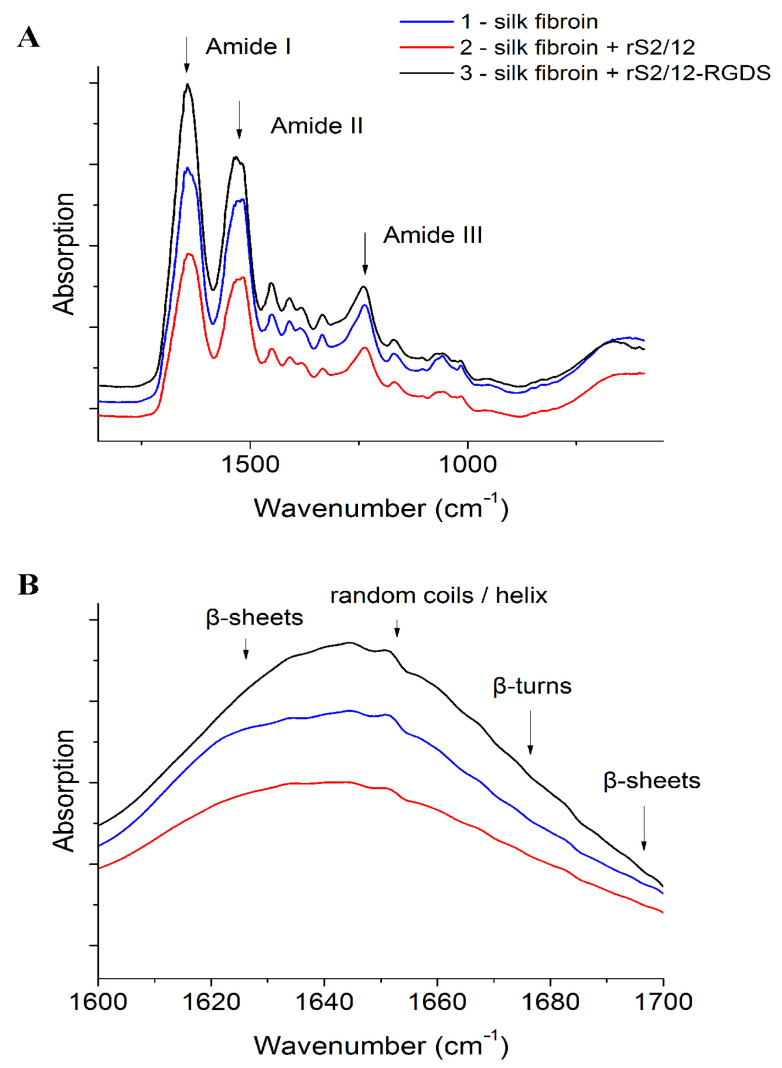
FTIR spectra of silk fibroin and spidroin-based scaffolds (**A**). The region of amide I (**B**) is shown separately.

The FTIR spectra analysis results of the scaffolds (Figure 2, Table 2) are characterized by the presence of bands that are typical for nitrogen compounds [30,31,32,33]: 3276 cm^−1^ (stretching vibrations of N-H bonds), 1639 cm^−1^ (with a shoulder in the region of 1650 cm^−1^, amide I band, stretching vibration C=O), 1526 cm^−1^ and its overtone at 3072 cm^−1^ (amide II band N-H bending vibrations and C-N stretching vibrations) and 1205–1280 cm^−1^ (amide III band N-H bending vibrations + C=O bending vibrations + C-C stretching vibrations). The results show that the introduction of spidroins into the composition of the fibroin-based scaffold does not significantly increase the content of β-sheets.

**Figure 3:**
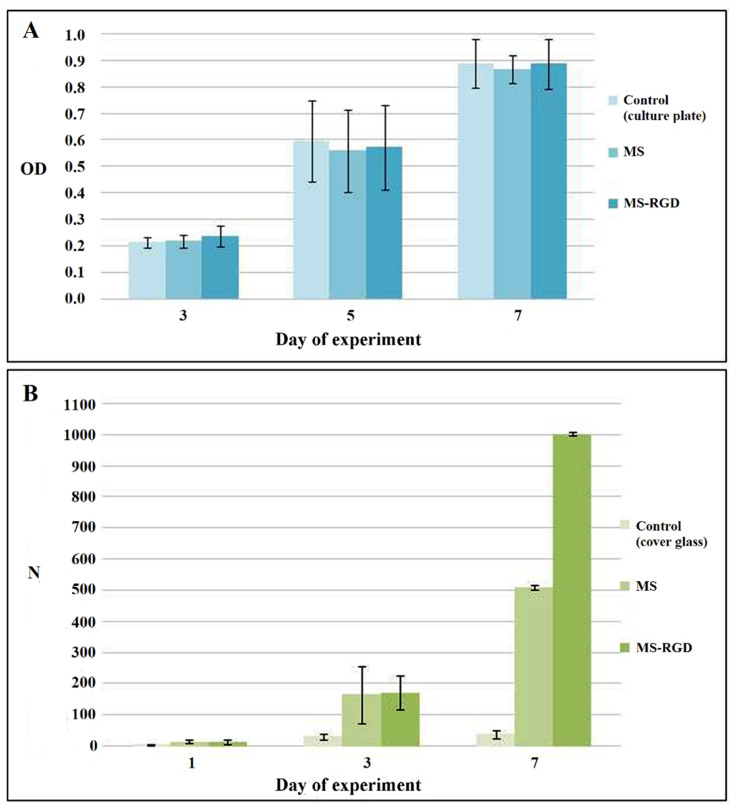
Results of experiments on the study of the cytotoxicity of the fabricated scaffolds and analysis of adhesion and proliferation of cells. (**A**): cytotoxicity on days 3, 5, and 7 of the experiment on the model of proliferation of mice 3T3 fibroblasts (MTT test—optical density at 540 nm of formazan solution in DMSO). The values of the standard deviation for 3 independent measurements are shown. (**B**): adhesion (1 day) and proliferative activity (3, 7 days) of Hep-G2 cells (cell quantity in microscopic field of 850 × 709 µm). Standard deviation values for 5 independent measurements are shown.

According to the cytotoxicity assay results, the fabricated scaffolds did not have a toxic effect on the cells, and the proliferative activity of the cells increased in experimental samples (Figure 3A).

An analysis of the cell adhesion and proliferation on the scaffolds was performed by counting the cell nuclei stained with DAPI fluorescent dye (Figure 3B). During the experiment, the scaffolds maintained cell adhesion at a level comparable to the control samples. No effect of the presence of the RGDS sequence on cell adhesion was found. This may be due to the fact that RGDS could be locked from cell receptors by fibroin molecules which do not contain such sequences. On the third day of the experiment, there was a significant increase in the proliferative activity of cells in the experimental constructions compared with the control samples. This can be explained both by the natural cytocompatible properties of fibroin and spidroins, due to their ability to provide a high level of cell proliferative activity, and by the porous structure of the constructions. On the seventh day of the experiment, the proliferative activity of cells in the scaffolds continued to increase; however, on constructions containing the RGDS sequence, it was significantly higher, which may be associated with the gradual degradation of fibers and the unlocking of the RGDS sequences, improving cell adhesion. Cell adhesion to the samples was uneven; cell migration into the deeper layers of the scaffolds was observed. In addition, there were areas of active cell proliferation into the samples without a cell monolayer.

**Figure 4:**
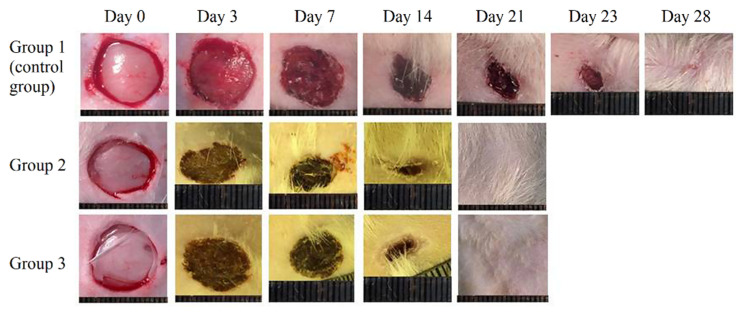
Images of wounds on days 0, 3, 7, 14, 21, 23, and 28 of the experiment. Complete healing of the wound in the control group occurred on the 40th day of the experiment (image not shown).

**Figure 5:**
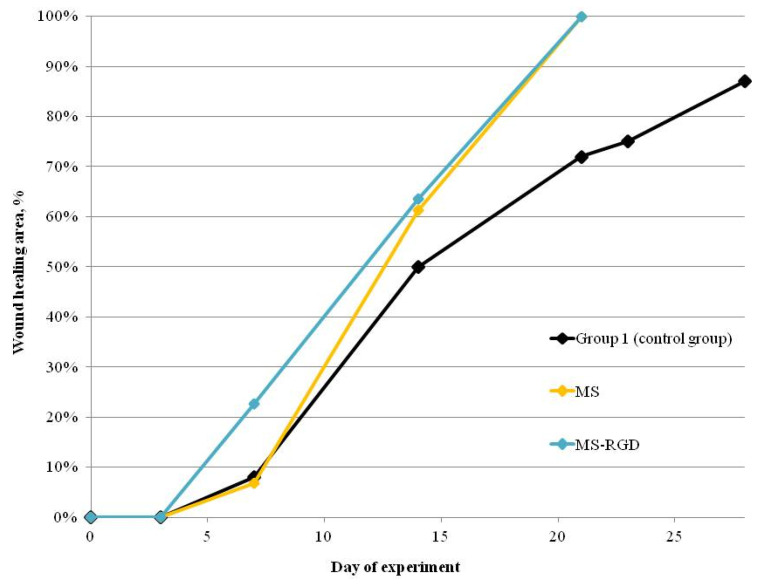
Wistar rat skin healing curves.

The fabricated scaffolds were successfully used as wound dressings for the full-thickness rat skin wound healing models (Figure 4). The constructions were used as wound dressings, which were positioned on the wound surface in a dry state. This positioning option does not lead to the formation of structural defects in scaffolds. The high-swelling property of the constructions ensures convenient implantation without causing additional damage, due to the absence of suture material fixated on the wound. According to the results of the experiments, the scaffolds accelerated the healing of the skin by 19 days compared with the control group, which is an indicator of the high regenerative potential of scaffolds (Table 3, Figure 5). At the first stages of wound healing, a similar effect was detected in the scaffolds containing the RGDS sequence as in vitro experiments, i.e., cells actively proliferated, leading to accelerated healing compared with other groups of animals.

**Figure 6:**
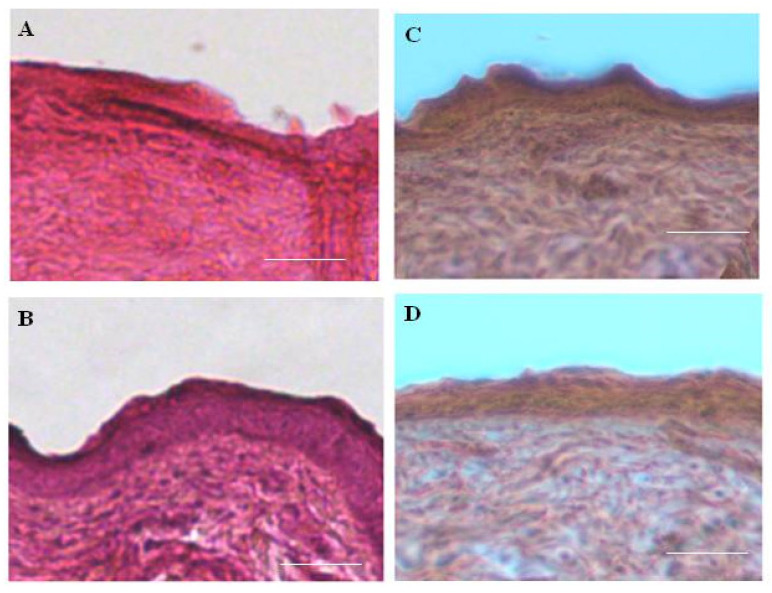
Images of histological sections of rat skin in the area of complete wound healing (A = 100%), stained with hematoxylin-eosin, magnification ×100, scale: 100 μm. (**A**): native rat skin (normal); (**B**): group 1 on the 40th day of the experiment; (**C**): group 2 on the 23rd day of the experiment; (**D**): group 3 on the 23rd day of the experiment.

A histological evaluation of skin samples in the area of complete wound healing (A = 100%) revealed the restoration of three layers of skin in all the experimental animals—epidermis, dermis, and hypodermis—which indicates successful wound healing (Figure 6). In this case, the morphology of sections of skin samples from the experimental animals does not differ from the morphology of sections of the native intact skin of a rat. No fragments of experimental scaffolds were found on the obtained sections, which indicates their complete biodegradation during skin healing. Furthermore, no foci of inflammation were identified, which is an important indicator characterizing the successful healing of skin. The results are in agreement with the data on the scarless healing of full-thickness skin wounds in rats obtained by a subcutaneous injection of microgels based on recombinant spidroin rS1/9 under the wound edges [34].

## 4. Conclusions

Within the framework of this study, two types of construction were fabricated based on silk fibroin containing recombinant spidroins rS2/12 and rS2/12-RGDS as composite additives correspondingly. These compositions were used for fabrication of the electrospun microfibrous scaffolds for the first time. The quantitative parameters of the structure of the electrospun scaffolds were estimated using the SEM method and the unique technology of SPNT. The novel data on micro- and nanostructure of electrospun scaffolds were obtained by SPNT technology. The fabricated constructions had no toxic effect on cells and were cytocompatible in vitro and biocompatible in vivo. The high regenerative potential of the constructions was shown: the rates of wound healing in both experimental groups were comparable with each other, and significantly (about two times) higher than one in the control group. Thus, the fabricated electrospun scaffolds can be considered as promising structures for promotion of wound healing.

## Figures and Tables

**Table 1:** Experimental groups with 5 animals in each group.

Group Number	Wound Dressing Description
1	- (control)
2	MS (silk fibroin + rS2/12)
3	MS-RGD (silk fibroin + rS2/12-RGDS)

**Table 2:** Deconvolution of the second derivative and the spectrum of scaffolds in the region of amide I depending on the composition of proteins.

Conformation	Content of Conformation, %		
	Fibroin Electrospun Scaffold	MS	MS-RGD
β-turn	22	27	22
β-sheet	42	46	33
Amorphous region	19	10	26
α-helix	17	27	19

**Table 3:** Dynamics of full-thickness skin wound healing in Wistar rats (standard deviation values for 5 independent measurements are shown). Sign “*” indicates statistically significant differences between the experimental groups and the control group.

Group Number	Wound Healing Area (%)						
	0 Day	3 Day	7 Day	14 Day	21 Day	23 Day	28 Day
1 (control group)	0	0	12.5 ± 1.0	50.0 ± 1.5	68.0 ± 1.5	75.0 ± 1.5	87.5 ± 1.7
2 (MS)	0	0	7.0 ± 1.2	61.0 ± 1.2 *	100 *		
3 (MS-RGDS)	0	0	23.0 ± 1.5 *	64.0 ± 0.6 *	100 *		

## Data Availability

The data presented in this study are available on request from the corresponding author.
